# Prospective associations between major depressive disorder, generalized anxiety disorder, fibromyalgia, and myalgic encephalomyelitis/chronic fatigue syndrome

**DOI:** 10.1017/S0033291725100603

**Published:** 2025-08-11

**Authors:** Nathaniel Stembridge Thomas, Michael C. Neale, Kenneth S. Kendler, Hanna M. van Loo, Nathan A. Gillespie

**Affiliations:** 1 Department of Psychiatry, Virginia Institute for Psychiatric and Behavioral Genetics, Richmond, VA, USA; 2 Department of Psychiatry, University of Groningen, Groningen, The Netherlands

**Keywords:** functional disorders, internalizing disorders, lifelines, longitudinal

## Abstract

**Background:**

Functional disorders (FDs) are associated with internalizing disorders (IDs). Studies investigating the nature of these associations over time are limited. We tested the direction of causation between measures of IDs (major depressive disorder [MDD], generalized anxiety disorder [GAD]) and FDs (fibromyalgia [FM] and myalgic encephalomyelitis/chronic fatigue syndrome [ME/CFS]) measured across two waves of longitudinal data (*N* = 108,034 and *N* = 73,590).

**Methods:**

The Lifelines Cohort Study is a large prospective population-based cohort study in the northeast of the Netherlands. We tested competing causal models for the longitudinal association between IDs and FDs and, to follow-up results from the model with all IDs and FDs, tested the direction of causation between MDD and FM.

**Results:**

FDs were more stable over time than IDs. Initial model comparisons support a bidirectional relationship between most IDs and FDs. Follow-up analyses support a unidirectional model where FM predicts MDD over time (*β* = 0.14, 95% confidence interval = [0.11, 0.18]), but not vice versa.

**Conclusions:**

The cross-time associations between ME/CFS, MDD, and GAD appear bidirectional (causal in both directions). Our results are consistent with, but not demonstrative of, a causal relationship from FM to MDD. The consequences of specific FDs vary, underscoring the value of studying these conditions as distinct constructs.

## Introduction

Functional disorders (FDs) are defined by the presence of somatic symptoms with unclear causes. FDs are diagnosed by the presence of particular somatic symptoms; for example, fibromyalgia (FM) for musculoskeletal pain (Bair & Krebs, 2020) and myalgic encephalomyelitis/chronic fatigue syndrome (ME/CFS) for severe fatigue and post-exertional malaise (Lim et al., 2020). FDs are prevalent (de Waal, Arnold, Eekhof, & van Hemert, 2004; Janssens, Zijlema, Joustra, & Rosmalen, 2015; Nimnuan, Hotopf, & Wessely, 2001), and are associated with reduced quality of life, increased disability, illness-related absence from work, and early retirement due to health problems (Joustra, Janssens, Bültmann, & Rosmalen, 2015), and are closely related to each other (Donnachie, Schneider, & Enck, 2020; Wessely, Nimnuan, & Sharpe, 1999). Although there is a strong association between FDs and internalizing disorders (IDs), the nature of this association remains largely unexplored. More insight into the shared and unique causes of these disorders could contribute to a better understanding of the etiology of both types of disorders.

Epidemiological studies have shown that FDs are comorbid with IDs, such as major depressive disorder (MDD) and generalized anxiety disorder (GAD) (Fishbain, Cutler, Rosomoff, & Rosomoff, 1997; Henningsen, Zimmermann, & Sattel, 2003; Kroenke, 2006; Yepez, Grandes, Talanki Manjunatha, Habib, & Sangaraju, 2022). In a previous study, we found substantial comorbidity between IDs (MDD and GAD) and FDs (FM and ME/CFS), with tetrachoric correlations ranging from 0.48 to 0.56 (Thomas et al., 2024). In terms of genetic etiology, FDs and IDs are heritable and share common genetic influences (Kato, Sullivan, Evengård, & Pedersen, 2009; Vassend, Røysamb, Nielsen, & Czajkowski, 2018), which likely accounts for some of the observed comorbidity. For example, one study reported heritability estimates ranging from 0.40 to 0.53 and genetic correlations ranging from 0.45 to 0.88 between fatigue symptoms, chronic musculoskeletal pain, and a composite of anxiety and depression (Vassend et al., 2018). The observed comorbidity and genetic covariance could be consistent with causal relationships between the disorders. Although temporal ordering is expected for causal relationships, it is possible that different thresholds for the two disorders could make the onset of one appear earlier than the other, even when causality is from the later diagnosis to the earlier one. Thus, there is a need to apply alternative methods of assessing causal relationships in nonexperimental settings.

Previous evidence for the direction of causation between IDs and FDs is conflicting. Two longitudinal studies have shown that MDD predicts future FM and chronic pain symptoms (Forseth, Husby, Gran, & Førre, 1999; Magni, Marchetti, Moreschi, Merskey, & Luchini, 1993). In contrast, two reports found that functional somatic symptoms, similar to those observed in FM and ME/CFS, predict future anxiety and depression (Nakao & Yano, 2006; van Boven et al., 2011). Other correlational studies have provided evidence of a bidirectional relationship between MDD and FM/chronic pain symptoms (Chang et al., 2015; Magni, Moreschi, Rigatti-Luchini, & Merskey, 1994). By bidirectional in a longitudinal context, we mean that (1) both Trait A at Time 1 predicts Trait B at Time 2 and (2) Trait B at Time 1 predicts Trait A at Time 2. This prediction is over and above any correlation arising from the correlation between Traits A and B at Time 1 and the within-trait autocorrelations (e.g. Trait A at Time 1 with Trait A at Time 2). Among the limitations of these studies was the failure to account for the autocorrelations in IDs and FDs that have been observed in previous studies (Hickie, Koschera, Hadzi-Pavlovic, Bennett, & Lloyd, 1999; Hoskin, Whipple, Nanda, & Vincent, 2018; Nivard et al., 2015). Prospective associations between conditions may be confounded with autoregressive associations within conditions; for example, earlier MDD may not predict later FM after accounting for earlier FM. In studies where within-trait stability is taken into account, functional somatic symptoms demonstrate modest prospective associations with depression and anxiety (Groen, van Gils, Emerencia, Bos, & Rosmalen, 2021; Janssens, Rosmalen, Ormel, van Oort, & Oldehinkel, 2010). One previous Mendelian randomization study found evidence that chronic multisite pain – one of the hallmark symptoms of FM – has a causal relationship with MDD (Johnston et al., 2019). Together, these studies suggest that the comorbidity between IDs and FDs may be explained by a causal effect of FDs on the development of IDs. However, previous studies have not examined cross-time, cross-condition associations between MDD, GAD, FM, and CFS together while simultaneously accounting for stability in each disorder over time.

In the current study, we assess the strength and direction of the cross-time associations between two IDs (MDD and GAD) and two FDs (FM and ME/CFS) in the large, population-based, longitudinal Lifelines cohort across two assessment waves (Scholtens et al., 2015; Sijtsma et al., 2022). We focus on these IDs and FDs because previous work indicates that associations between these conditions are particularly large (Thomas et al., 2024).

**Figure 1.**
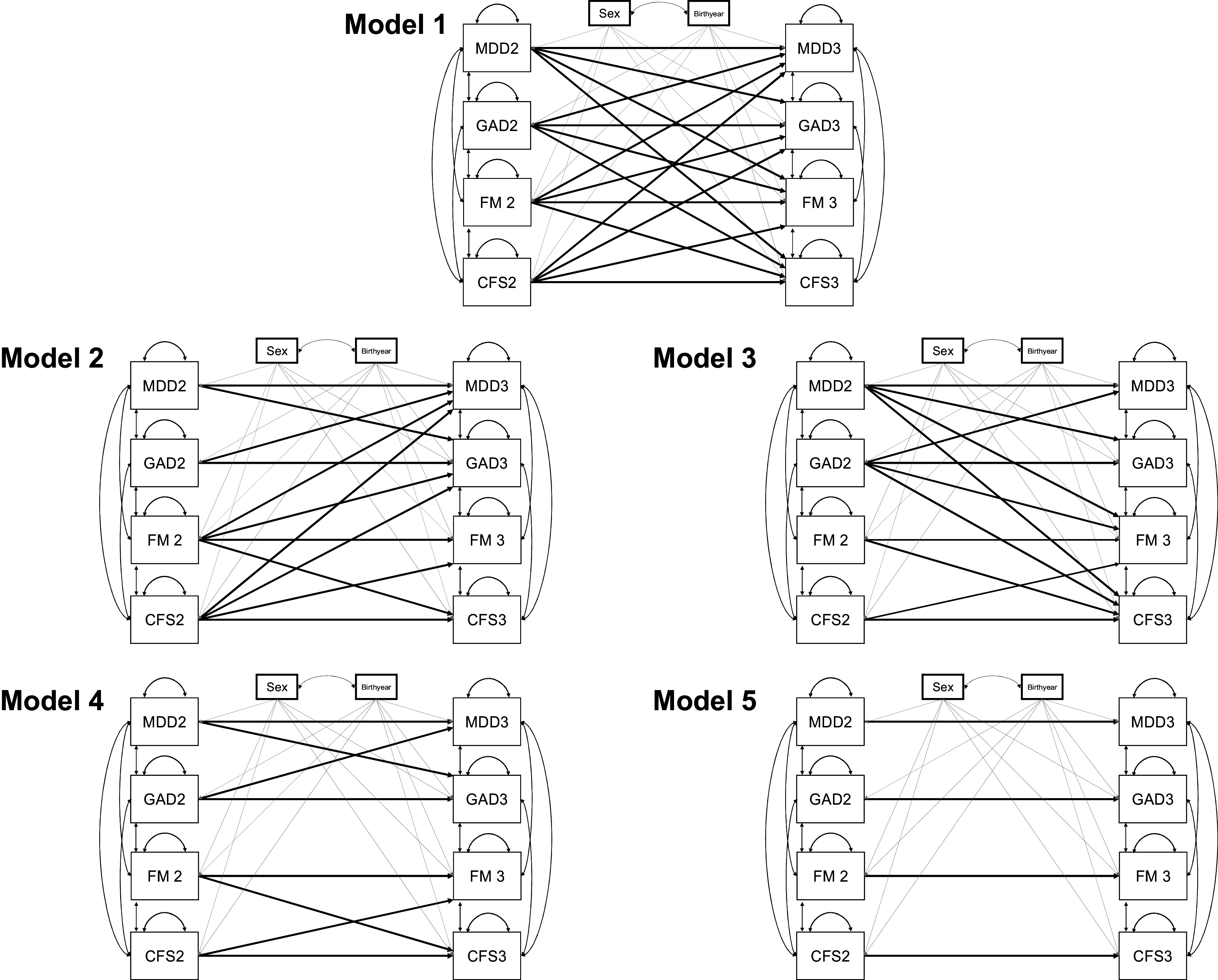
Factor score path analysis models of internalizing disorders (IDs: MDD and GAD) and functional disorders (FDs: FM and ME/CFS) Path diagrams for five models of the cross-time association between IDs (MDD and GAD) and FDs (FM, ME/CFS). All cross-time effects between IDs and FDs at Wave 2 and Wave 3 are estimated in Model 1. Model 2 includes only the cross-time effects from FDs at Wave 2 to IDs at Wave 3. Model 3 mirrors this specification, estimating cross-time effects from IDs at Wave 2 to FDs at Wave 3. Model 4 includes cross-time effects of IDs at Wave 2 on IDs at Wave 3 and FDs at Wave 2 on FDs at Wave 3. Finally, Model 5 includes only autoregressive paths from Wave 2 to Wave 3 within diagnosis. Note: FM, ‘fibromyalgia’; GAD, ‘generalized anxiety disorder’; MDD, ‘major depressive disorder’; ME/CFS, ‘myalgic encephalomyelitis/chronic fatigue syndrome’.

Our first goal was to test five competing models concerning the nature of the longitudinal associations between IDs (MDD and GAD) and FDs (FM and ME/CFS) across two assessment waves using all available data. We use the term “bidirectional” throughout this article to refer to a pattern of longitudinal association where Trait A at Time 1 predicts Trait B at Time 2 and Trait B at Time 1 predicts Trait A at Time 2. We use the term “unidirectional” to refer to instances where Trait A at Time 1 predicts Trait B at Time 2, but Trait B at Time 1 does not predict Trait A at Time 2. As illustrated in Figure 1, the five models are as follows: (1) a fully bidirectional relationship between all conditions, (2) a unidirectional model where FDs predict IDs, (3) a unidirectional model where IDs predict FDs, (4) a model where IDs predict IDs, and FDs predict FDs, and (5) a model where only autoregressive paths were included. Our second goal was to evaluate evidence for specific unidirectional associations between MDD, GAD, FM, and ME/CFS that emerged from the analysis of factor scores. Since one pair of conditions demonstrated asymmetrical cross-time effects (MDD and FM), the prospective association between MDD and FM was assessed in a follow-up analysis using a cross-lagged structural equation model (SEM).

## Methods

### Sample

Lifelines is a multidisciplinary prospective population-based cohort study examining, in a three-generation design, the health and health-related behaviors of 167,729 persons living in the North of the Netherlands. It employs a broad range of investigative procedures in assessing the biomedical, sociodemographic, behavioral, physical, and psychological factors that contribute to the health and disease of the general population, with a special focus on multimorbidity and complex genetics (Scholtens et al., 2015; Sijtsma et al., 2022; Stolk et al., 2008). Between 2006 and 2013, an index population aged 25–49 years was recruited via participating general practitioners (GPs). Exclusion criteria were limited life expectancy, severely impaired decisional capacity, and inability to visit the GP, complete questionnaires, or understand the Dutch language. Participants who gave written informed consent were asked to indicate whether family members (partner, parents, parents-in-law, and children) could be invited to participate and were requested to provide contact details. In addition, adults could self-register via the Lifelines website. In total, 49% of the included participants were invited through their GP, 38% were recruited via participating family members, and 13% self-registered. Baseline data were collected for 167,729 participants (age range 6 months–93 years; 91.2% adults). Lifelines has ~97,000 female (58%) and ~ 71,000 male participants (42%).

A follow-up assessment was completed by 123,061 participants (73%) between 2014 and 2019 (Wave 2). Wave 3 data collection started in 2020 and is ongoing. Currently, ~50% of the initial sample has responded to Wave 3. Our analyses here rely on data from Wave 2 and Wave 3, where self-reported diagnostic criteria for IDs and FDs were both available. Up to 3 years could separate the assessment of IDs and FDs within a wave, meaning that a participant may have multiple values for age for each different ID and FD diagnosis within a wave. We removed participants with an interval between assessments greater than 6 months to reduce the difference in the timing of assessments within the wave.

### Measures

#### Internalizing disorders

MDD symptoms in the past 2 weeks and GAD symptoms in the past 6 months were assessed using the Mini-International Neuropsychiatric Interview (MINI; Sheehan et al., 1998) as a computerized questionnaire at the research site. Items on the MINI conform to the Diagnostic and Statistical Manual of Mental Disorders, 4th Edition (DSM-IV), and the International Classification of Diseases, 10th Revision criteria. The symptoms of MDD and GAD were used as binary items for factor analysis. Diagnoses of MDD and GAD were established according to DSM-IV-Text Revision criteria, except that we did not consider disability criteria, as these were not assessed in the MINI (Loo et al., 2023).

#### Functional disorders

The diagnostic criteria for current FM were assessed using the 2010 American College of Rheumatology (ACR) criteria (Wolfe et al., 2010). Participants were asked to indicate in which of the 19 mentioned body areas they had experienced pain during the last week using the Widespread Pain Index (WPI). The Symptom Severity (SS) scale was calculated based on the severity of fatigue, cognitive symptoms, waking unrefreshed, and somatic symptoms participants reported. The severity of fatigue and cognitive symptoms in the past 2 weeks was determined using items from the Checklist Individual Strength (Vercoulen et al., 1994). An additional item that determined to what extent participants are waking unrefreshed was added. To determine the level of somatic symptoms in the past week, the 12-item Somatization scale of the Symptom Checklist-90 (SCL-90 SOM) was used (Arrindell & Ettema, 2003). To meet the ACR diagnostic criteria, participants are required to have a WPI score ≥ 7 and an SS-scale score ≥ 5, or a WPI score of 3–6 and an SS-scale score of ≥9. In line with the diagnostic criteria, we scored the WPI ordinally for factor analysis (<3 = 0, 3–6 = 1, and 7+ = 2). We applied a similar approach for the scoring of the SS-scale (<5 = 0, 5–8 = 1, and 9+ = 2). The final FM criteria indicator was a binary item derived from the diagnostic criteria that participants had to indicate that they experienced pain symptoms for at least 3 months.

The diagnostic criteria for current ME/CFS were assessed using the 1994 Centers for Disease Control and Prevention (CDC) criteria (Fukuda et al., 1994). To meet the CDC diagnostic criteria, participants had to indicate (1) that they had experienced chronic fatigue for 6 or more months, and (2) that the fatigue significantly interfered with daily activities and work in the past 6 months. In addition, (3) the participant had to report concurrently four or more of the eight mentioned additional symptoms in the past 6 months. Each of these three criteria was used as binary items for factor analysis.

#### Covariates

Covariates included self-reported biological sex (scored as a binary outcome) and birth year.

### Analysis plan

To assess the direction of cross-time associations between two IDs (MDD and GAD) and two FDs (FM and ME/CFS), our analysis plan consisted of three steps, described in greater detail below. First, we estimated measurement models and derived factor scores for the common factors of the symptoms of each condition at each wave. Second, we conducted path analysis of the factor scores for MDD, GAD, FM, and ME/CFS across time, estimating the longitudinal associations between all conditions simultaneously. Third, we estimated an exploratory SEM to further investigate an emergent, asymmetrical longitudinal association between MDD and FM. Specific FDs and IDs for further analyses were identified by comparing the magnitude of path coefficients in the path analysis of factor scores. For example, if the effect of an FD at Wave 2 on an ID at Wave 3 was significantly different from the effect of an ID at Wave 2 on an FD at Wave 3, we modeled the longitudinal association between the two conditions in a follow-up analysis using cross-lagged SEM. All path analyses and SEM modeling were conducted using the OpenMx software package Version 2.21.8 (Neale et al., 2016) in R Version 4.2.2 (R Core Team, 2022).

#### Full Information Maximum Likelihood (FIML) measurement models and factor scoring

The common factors for the four conditions across two waves comprise of a total of 40 binary indicators and 4 ordinal indicators, precluding estimation of the measurement models and cross-time associations for all conditions in one model. We used factor scores for this step in the analysis plan to estimate the prospective association between all conditions simultaneously. A common factor model was fitted to the symptoms of each condition at each wave by FIML. Supplementary Figures S1–S8 present the measurement models for IDs and FDs. Common factor means and variances were fixed at 0 and 1, respectively. Binary variables were modeled by fixing the mean to 0, the variance to 1, and estimating the threshold as a free parameter. Ordinal variables were modeled by fixing the first two thresholds to 0 and 1 and estimating the mean and variance as free parameters, as discussed in Mehta, Neale, and Flay (2004). To test for measurement invariance by sex, three multigroup models were fit for each measurement model: (1) equal thresholds and factor loadings across sex, (2) equal thresholds and unequal factor loadings across sex, and (3) unequal thresholds and unequal factor loadings across sex. Factor scores were estimated by FIML to accommodate missing symptom data for participants with valid data for at least two items in each measurement model.

#### Factor score path analysis and model comparisons

Factor scores were estimated separately for each individual in the sample, using the maximum likelihood parameter estimates from the full information factor analyses. All models included the within-time covariances of the Wave 2 and Wave 3 factor scores. Sex and birth year were included as fixed-effect covariates. We tested five competing models to explain the within- and cross-temporal relationship between IDs and FDs – (1) *Fully bidirectional*: All IDs and FDs at Wave 2 predict all IDs and FDs at Wave 3; (2) *FD2 to ID3*: Only FDs at Wave 2 predict IDs at Wave 3; (3) *ID2 to FD3*: Only IDs at Wave 2 predict FDs at Wave 3; (4) *ID2 to ID3 and FD2 to FD3*: From Wave 2 to Wave 3, IDs predict IDs, and FDs predict FDs; and (5) *Autoregression*: Only autoregressive paths are included (see Figure 1).

The significance of cross-time associations was determined using the change in the minus two Log-Likelihood (∆-2LL), which, under certain regularity conditions (Steiger, Shapiro, & Browne, 1985), follows a *χ*^2^-distribution with degrees of freedom equal to the difference in the number of parameters in the two models. We also considered Akaike’s Information Criterion (AIC), which balances model complexity and goodness of fit. We report the change in AIC and the likelihood-ratio test (LRT) *p*-value for each comparison. Thus, the LRT indicates whether the parameter constraints applied in each reduced model resulted in a statistically significant decrease in model fit relative to the fully bidirectional model, while the change in AIC assesses the efficiency with which the parameters describe the data.

#### Exploratory DWLS cross-lagged SEM

We estimated a cross-lagged SEM to assess the associations between specific IDs and FDs that emerged from the analysis of factor scores. We tested the following three models: (1) a bidirectional relationship between the ID and the FD, (2) a unidirectional model where the ID predicts the FD, and (3) a unidirectional model where the FD predicts the ID. We used diagonally weighted least squares (DWLS) to accommodate the large number of binary symptom indicators underlying the ID common factors. Model fit was assessed using pseudo-AIC, defined as the sum of the *χ*^2^-statistic and two times the number of parameters, and the Satorra–Bentler adjusted *χ*^2^-difference test (Satorra & Bentler, 2001).

## Results

In order to characterize the analytic sample as a whole, we limited the dataset to participants who had non-missing data for at least two symptoms/criteria of any condition (Wave 2 *N* = 108,034 and Wave 3 *N* = 73,590). The mean age was 49.3 (standard deviation [SD] = 13.0, range = 18.0–96.0) years, and 63,301 (58.6%) participants were female in Wave 2. The mean age was 55.1 (SD = 12.8, range = 18.0–96.3) years, and 43,153 (58.6%) participants were female in Wave 3. ID and FD symptom frequencies at Wave 2 and Wave 3 are presented in Table 1.

**Table 1.** MDD, GAD, ME/CFS, and FM symptom/criteria frequencies in Wave 2 and Wave 3

	Wave 2			Wave 3		
	No	Yes	%	No	Yes	%
MDD						
1. Consistently depressed	87,638	3,746	4.1%	67,715	2,420	3.5%
2. Much less interested in most things	86,364	5,020	5.5%	66,420	3,727	5.3%
3. Appetite noticeably changed	82,771	8,613	9.4%	62,944	7,153	10.2%
4. Trouble sleeping nearly every night	70,494	20,890	22.9%	53,508	16,715	23.8%
5. Move slowly OR feel restless	86,455	4,927	5.4%	66,710	3,455	4.9%
6. Tired	80,035	11,349	12.4%	61,282	8,939	12.7%
7. Feel worthless or guilty	88,490	2,894	3.2%	68,261	2,000	2.8%
8. Difficulty concentrating	84,407	6,977	7.6%	65,010	5,246	7.5%
9. Repeatedly consider self-harm, feel suicidal	90,385	999	1.1%	69,612	638	0.9%
GAD						
1. Excessive, daily, and difficult to control	85,299	6,085	6.7%	65,914	4,435	6.3%
2. Restless	76,581	14,803	16.2%	59,496	10,726	15.3%
3. Tense	68,339	23,045	25.2%	54,063	16,154	23.0%
4. Tired, weak, or exhausted easily	69,227	22,157	24.2%	52,768	17,451	24.9%
5. Difficulty concentrating	77,345	14,039	15.4%	59,665	10,550	15.0%
6. Irritable	74,875	16,509	18.1%	58,140	12,063	17.2%
7. Difficulty sleeping	64,018	27,366	29.9%	47,531	22,681	32.3%
ME/CFS						
1. Fatigue for 6 or more months	33,276	28,134	45.8%	21,953	19,990	47.7%
2. Fatigue interferes with daily activities	78,908	10,082	11.3%	52,471	6,827	11.5%
3. Four or more additional symptoms	82,589	6,122	6.9%	54,089	5,222	8.8%
FM						
1. Pain symptoms present for 3+ months	49,834	39,389	44.1%	31,631	27,774	46.8%
2. WPI score	<3	3–6	7+	<3	3–6	7+
	54,833	26,767	7,727	37,457	17,194	4,764
3. SS score	<5	5–8	9+	<5	5–8	9+
	60,779	25,906	2,717	42,502	15,383	1,557

Abbreviations: FM, fibromyalgia; GAD, generalized anxiety disorder; MDD, major depressive disorder; ME/CFS, myalgic encephalomyelitis/chronic fatigue syndrome; SS, symptom severity; WPI, Widespread Pain Index.

### Measurement models

The measurement models for MDD (Wave 2: root mean square error of approximation [RMSEA] = 0.020, 95% confidence interval [CI] = [0.019, 0.021], Comparative Fit Index (CFI) = 0.98, Tucker-Lewis Index (TLI) = 0.97 and Wave 3: RMSEA = 0.013, 95% CI = [0.012, 0.014], CFI = 0.99, TLI = 0.98) and GAD (Wave 2: RMSEA = 0.039, 95% CI = [0.038, 0.041], CFI = 0.98, TLI = 0.96 and Wave 3: RMSEA = 0.037, 95% CI = [0.035, 0.038], CFI = 0.97, TLI = 0.96) provided a good fit to the data. The measurement models for FM and ME/CFS were saturated, so fit statistics could not be calculated. See Supplementary Figures S1–S8 for path diagrams of the measurement models for IDs and FDs.

We identified statistically significant differences in factor loadings and thresholds across sex. Estimates from the measurement models that allowed for sex differences are included in Supplementary Table S1. To assess if this statistical difference in the measurement models would result in a meaningful difference in the resulting factor scores, we calculated factor scores from (1) a model with equal thresholds and loadings and (2) a model with unequal thresholds and loadings. Correlations between the factor scores were near unity in all cases (*r* = 0.89–0.99). Tests of measurement invariance by sex are presented in Supplementary Table S2, and the correlations between the factor scores from full-sample and sex-stratified versions of the analysis for each condition and wave are presented in Supplementary Table S3. Given the large correlations between the factor scores, we proceeded with the simpler model that assumed equivalence of loadings and thresholds between sexes.

### Longitudinal associations between IDs and FDs: Path analysis and model comparisons

**Figure 2.**
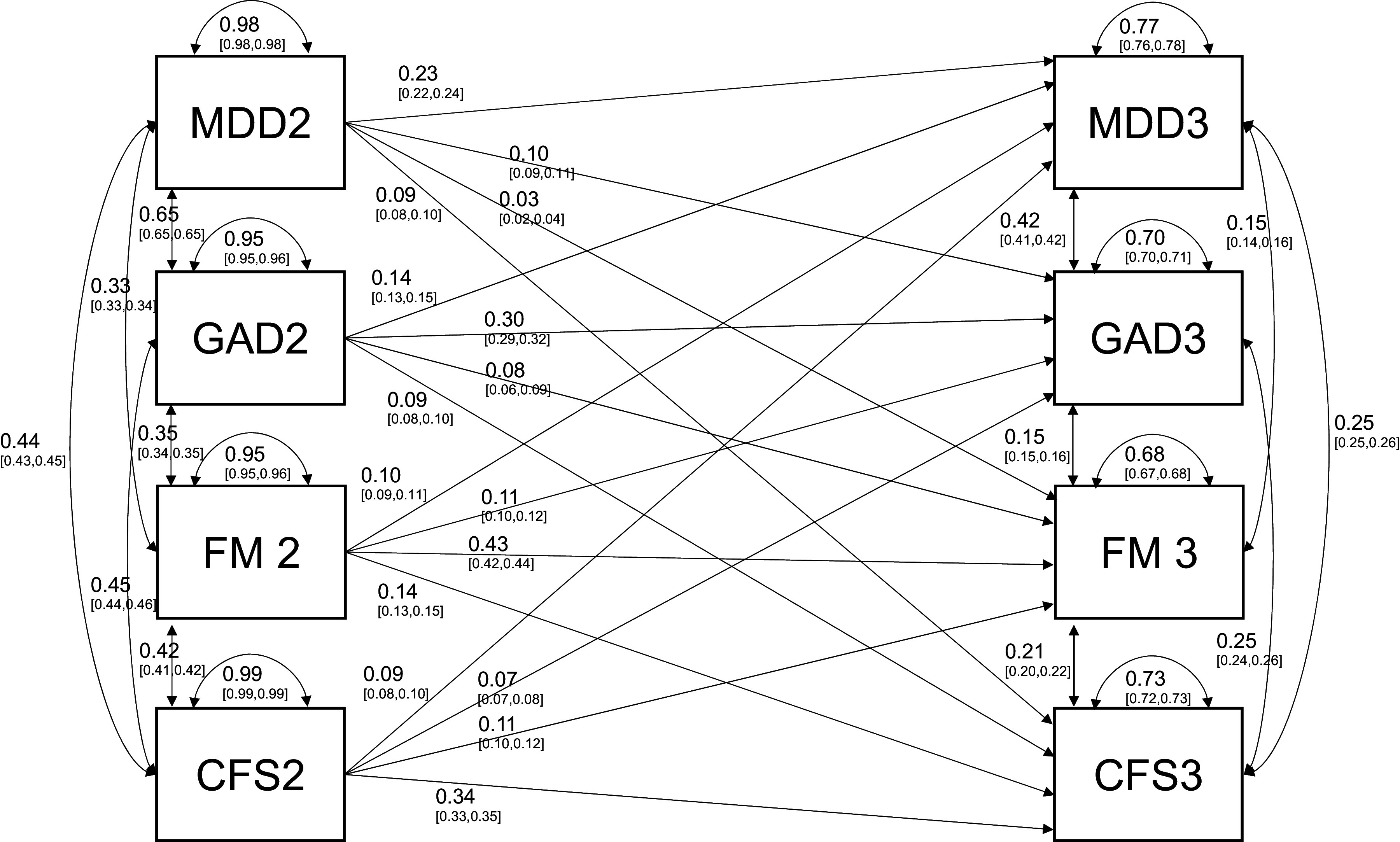
Standardized coefficient estimates from the fully bidirectional path analysis of the relationship between internalizing disorders and functional disorders over time. Coefficient estimates from the best-fitting fully bidirectional model of IDs and FDs fit by FIML. All coefficient estimates are standardized and 95% confidence intervals are presented in brackets. Note: FM, ‘fibromyalgia’; GAD, ‘generalized anxiety disorder’; MDD, ‘major depressive disorder’; ME/CFS, ‘myalgic encephalomyelitis/chronic fatigue syndrome’.

The *Fully bidirectional* model provided the best fit to the data. The *FD2 to ID3* model provided a better fit than the *ID2 to FD3* model. These results suggest that the longitudinal associations between most IDs and FDs are bidirectional, with larger associations from earlier FDs to later IDs. Model fit declined further in the *ID2 to ID3 and FD2 to FD3* model and the *Autoregression* model, which do not allow for longitudinal associations between IDs and FDs. All LRTs comparing model fits were statistically significant (*p* < 0.001), indicating that all of the model constraints tested here resulted in a significant decrease in model fit. Table 2 summarizes the comparisons between the five competing models testing the associations between IDs and FDs over time, which are illustrated in Figure 1. Standardized coefficient estimates from all five models are presented in Supplementary Table S4. A diagram of the standardized coefficient estimates and 95% CIs for the best-fitting *Fully bidirectional* model is presented in Figure 2.

**Table 2.** Factor score path analysis to test competing hypotheses regarding the direction of causation between IDs and FDs

Model	ep	-2LL	df	∆-2LL	∆df	*p*	AIC
1. Fully bidirectional	64	1,253,414	943,153				1,253,542
2. FD2 to ID3	60	1,254,551	943,157	1137	4	<0.001	1,254,671
3. ID2 to FD3	60	1,254,879	943,157	1465	4	<0.001	1,254,999
4. ID2 to ID3 and FD2 to FD3	56	1,256,724	943,161	3310	8	<0.001	1,256,836
5. Autoregression	52	1,261,100	943,165	7686	12	<0.001	1,261,204

*Note:* Comparisons between the saturated (1) Fully bidirectional (reference) model and the four nested sub-models: (2) the unidirectional FDs at Wave 2 to IDs at Wave 3; (3) the unidirectional IDs at Wave 2 to FDs at Wave 3; (4) IDs at Wave 2 to IDs at Wave 3 and FDs at Wave 2 to FDs at Wave 3; and (5) Autoregression. Model numbers in the table correspond to the path diagrams included in Figure 1.

Abbreviations: AIC, Akaike Information Criteria; ep, number of estimated parameters; FD, functional disorder, ID, internalizing disorder; −2LL, −2 × log-likelihood; ∆-2LL, change in −2 × log-likelihood, ∆df, change in degrees of freedom.

In the best-fitting *Fully bidirectional* model, the autoregressive associations were the largest cross-time associations for all conditions (*β* = 0.23–0.43). The effects of Wave 2 IDs on Wave 3 IDs (*β*s: 0.10–0.14) and Wave 2 FDs on Wave 3 FDs (*β*s: 0.11–0.14) were larger than other cross-condition, cross-time associations. The effects of Wave 2 FD on Wave 3 ID were modest (*β*s: 0.07–0.11), but somewhat larger than the effects of Wave 2 IDs on Wave 3 FDs (*β*s: 0.03–0.09). The association between Wave 2 MDD and Wave 3 FM (*β* = 0.03, 95% CI = [0.02, 0.04]) was smaller than that between Wave 2 FM and Wave 3 MDD (*β* = 0.10, 95% CI = [0.09, 0.11]). The CIs for these two *β* estimates did not overlap. Additionally, model fit deteriorated significantly when the two coefficients were fixed to equality in an otherwise-identical model 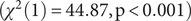 , indicating a significant difference in the size of the cross-time effects between MDD and FM. To examine this asymmetrical cross-time relationship more closely, we estimated a cross-lagged SEM between these two conditions.

### Longitudinal associations between MDD and FM: Exploratory DWLS SEM

We found evidence of one asymmetrical relationship across time in the path analysis of factor scores: The association between Wave 2 FM and Wave 3 MDD was significantly larger than the association between Wave 2 MDD and Wave 3 FM 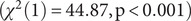 . To explore further the longitudinal association between these two diagnoses, we fitted an exploratory cross-lagged SEM between the Wave 2 and Wave 3 MDD common factors and the Wave 2 and Wave 3 FM common factors to test the direction of causation.

Specifically, we tested three models for the association between MDD and FM over time: (1) *Bidirectional*, where MDD and FM at Wave 2 predict both MDD and FM at Wave 3; (2) only MDD predicts FM over time (*MDD2 to FM3*), and (3) only FM predicts MDD over time (*FM2 to MDD3*). When covariates were included in the model (Supplementary Figures S9–S11), we observed evidence of optimization failure. Therefore, we removed covariates and constrained factor loadings to be equal across time to achieve model convergence. Our justification for removing covariates is provided in Supplemental Note 1, including a sensitivity analysis to determine the significance of model covariates to our results (Supplementary Figure S12).

**Figure 3.**
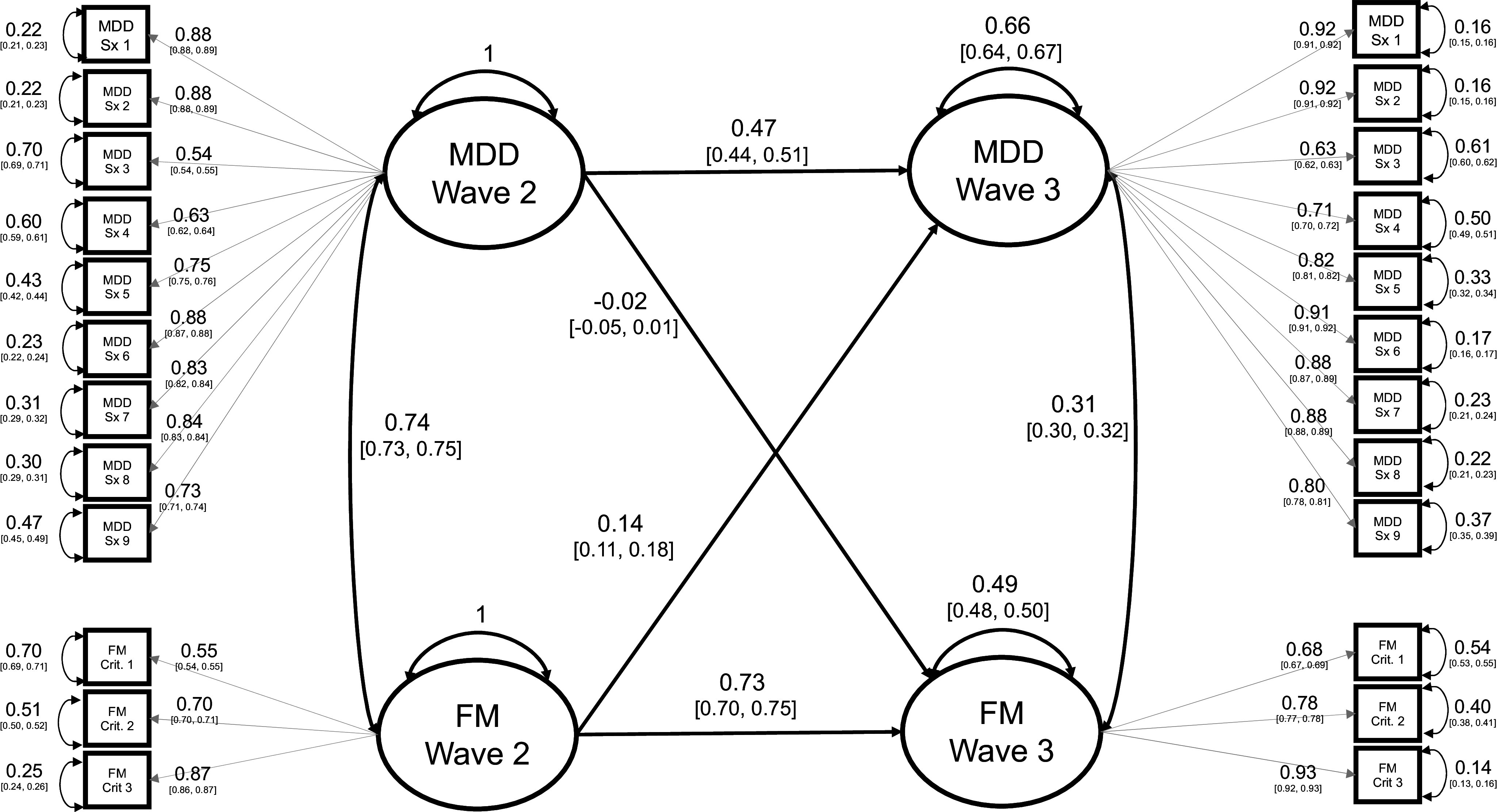
Cross-lagged SEM of the relationship between major depressive disorder (MDD) and fibromyalgia (FM) between Wave 2 and Wave 3 (Bidirectional). Coefficient estimates from the Bidirectional model of MDD and FM fit by diagonally weighted least squares. Model comparisons between the Bidirectional model, the MDD to FM model, and the FM to MDD model are presented at the bottom of the figure. Symptom indicators are numbered in alignment with Table 1. All coefficient estimates are standardized and 95% confidence intervals are presented in brackets. Note: chisq, ‘chi-squared’, df, ‘degrees of freedom’, ep, ‘number of estimated parameters’, FM, ‘fibromyalgia’, MDD, ‘major depressive disorder’, pseudo-AIC, ‘pseudo Akaike Information Criteria (sum of the *χ*^2^-statistic and two-times the number of parameters)’, SB Diff, ‘Satorra–Bentler adjusted *χ*^2^- difference’.

We then refit the three models without covariates: *Bidirectional* (Figure 3), *MDD2 to FM3* (Supplementary Figure S13) and *FM2 to MDD3* (Supplementary Figure S14). The cross-time coefficient estimates were larger in the SEM compared to the factor score model, reflecting the benefits of accounting for measurement error in a single-step latent variable model compared to the two-step approach via factor scoring. Again, the autoregressive associations were the largest cross-time effects in the model, and greater stability was observed in FM than MDD (FM2 to FM3: *β* = 0.73, 95% CI = [0.70, 0.75] and MDD2 to MDD3: *β* = 0.47, 95% CI = [0.44, 0.51]). Pseudo-AICs indicate that the *FM2 to MDD3* model provided a better fit to the data than the *MDD2 to FM3* model. The difference between the pseudo-AIC of the *FM2 to MDD3* model (pseudo-AIC = 22,494) and the *Bidirectional* model (pseudo-AIC = 22,495) was small; however, the path coefficient for the effect of Wave 2 MDD on Wave 3 FM was not significantly different than 0 (*β* = −0.02, 95% CI = [−0.05, 0.01]). Relatedly, the Satorra–Bentler adjusted *χ*^2^-difference test indicates that constraining the path from Wave 2 MDD to Wave 3 FM to zero (model *FM2 to MDD3*) did not result in a statistically significant reduction in model fit relative to the *Bidirectional* model 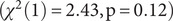 . By contrast, the cross-time association between Wave 2 FM and Wave 3 MDD was significantly greater than 0 (*β* = 0.14, 95% CI = [0.11, 0.18]). Model fit deteriorated significantly when the cross-time associations between MDD and FM were constrained to equality 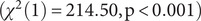 , indicating that the effect of earlier FM on later MDD is significantly larger than the effect of earlier MDD on later FM. These results are consistent with, but not demonstrative of, a causal relationship from earlier FM to later MDD.

## Discussion

This study tested a series of five models for the longitudinal association between IDs and FDs. We then evaluated evidence for a unidirectional association from FM to MDD. The longitudinal associations between IDs and FDs were bidirectional, with the exception of MDD and FM, where FM predicts MDD over time but not vice versa.

### Path analysis and SEM modeling

The comparison of path models with factor scores supported a fully bidirectional relationship between IDs and FDs over time. The autoregressive effects were the largest cross-time associations and varied between conditions. Cross-time effects within IDs (MDD2 to GAD3 and GAD2 to MDD3) and FDs (FM2 to ME/CFS3 and ME/CFS2 to FM3) were larger than the cross-time effects between IDs and FDs, although our results also support substantial prospective associations between IDs and FDs. The association between Wave 2 FM and Wave 3 MDD was larger than the association between Wave 2 MDD and Wave 3 FM. By contrast, the effect of Wave 2 ME/CFS on Wave 3 MDD was comparable to the other cross-condition, cross-time effects observed in the model. The correlates of specific FD conditions, such as FM and ME/CFS, vary despite the substantial comorbidity between them. Our results underscore the value of studying these conditions as related but distinct constructs.

In the follow-up analysis of MDD and FM, we found additional evidence to support the asymmetrical effect of Wave 2 FM on Wave 3 MDD. Our results are similar to a previous study that reports a modest cross-time effect of functional somatic symptoms on MDD (Janssens et al., 2010) and suggest that MDD does not predict the development of future FM above the effect of earlier FM. These results are compatible with, but not demonstrative of, causation from earlier FM to later MDD. One previous Mendelian randomization study found similar results, which support causation from chronic multisite pain to MDD (Johnston et al., 2019). FDs are associated with reduced quality of life, increased disability, illness-related absence from work, and early retirement due to health problems (Joustra et al., 2015), all of which represent possible mechanisms of a putative causal effect of FM on MDD. Future studies that investigate the role of functional limitations in the association between FM and MDD are recommended. The cross-trait, cross-time effect was modest relative to the stability in each condition over time. FM was found to be more stable than MDD. The autoregressive path coefficients in the follow-up analysis were substantially larger than the estimates from the factor score path analysis, suggesting that the factor score model may have underestimated these effects.

### Limitations

We note several limitations of the current study. First, we used factor scoring for the model that included all IDs and FDs. While this facilitated simultaneous analysis of all conditions across time, this approach assumes that the factor scores are measured without error, which they are not. Future work may consider methods that incorporate measurement error into the analysis of factor scores (Lai & Hsiao, 2022). Second, we used an ordinal version of the WPI and SS-scale scores to define the FM common factors. This approach approximates the ACR diagnostic criteria for FM but may have resulted in loss of information relative to the continuous scale scores. Third, IDs and FDs were not assessed simultaneously within a wave. We removed participants where differences were greater than 6 months, but the remaining smaller differences in the timing of assessments may still introduce error into the associations between diagnoses within waves, reducing the estimated within-wave associations. Fourth, both waves of data included here are follow-up assessments, and data collection for Wave 3 in Lifelines is not yet complete. Systematic differences may exist between participants who have responded in follow-up assessments and the initial sample, limiting the generalizability of our findings. We estimated tetrachoric correlations to examine differential attrition, including (1) ID symptoms at Wave 1 with missingness at Wave 2 and (2) ID symptoms/FD criteria at Wave 2 with missingness at Wave 3 (Supplementary Table S6). FD criteria were not assessed at Wave 1. Most ID symptoms and FD criteria were positively associated with subsequent missingness, with significant correlations ranging from 0.03 to 0.13, indicating that participants with more ID symptoms and FD criteria were less likely to respond to follow-up assessments. Thus, our analytic sample may not be representative of the overall Lifelines sample. Fifth, the Lifelines sample includes relatives, and we did not model the correlation between family members in order to reduce model complexity and facilitate model convergence. The standard errors presented here are somewhat smaller than they would be in a model that accounts for familial relationships, although the large overall sample size helps to mitigate this concern. Relatedly, we also did not account for genetic/familial noncausal mechanisms that may drive the observed temporal associations, such as pleiotropy or correlated environmental risks. Future studies that apply causal inference methods to account for these confounding factors will be a valuable addition to the literature. Finally, our analyses focus on only two FDs, that is, FM and ME/CFS. Our results may not generalize to other FDs, such as irritable bowel syndrome, which has been shown to have smaller covariance with IDs compared to FM and ME/CFS (Thomas et al., 2024).

## Conclusions

In the current study, we assessed cross-time associations between MDD, GAD, FM, and ME/CFS in a large population-based longitudinal cohort across two waves of data. FDs were more stable over time compared to IDs. We found evidence of bidirectional associations between most IDs and FDs. In our follow-up analysis, asymmetrical cross-time associations suggest that FM predicts MDD over time, but not vice versa. Future studies that apply methods for causal inference (Castro-de-Araujo et al., 2023; Ohlsson & Kendler, 2020; Smith & Ebrahim, 2003) for the relationship between MDD and FM is recommended. Refining our understanding of causal relationships between, or shared risk factors for, IDs and FDs will be key to understanding the etiology of these conditions.

## Supporting information

Thomas et al. supplementary material 1 Thomas et al. supplementary material

Thomas et al. supplementary material 2 Thomas et al. supplementary material

## Data Availability

Data may be obtained from a third party and are not publicly available. Researchers can apply to use the Lifelines data used in this study. More information about how to request Lifelines data and the conditions of use can be found on their website (https://www.lifelines.nl/researcher/how-to-apply).
