# Supplementary material for: Prospective associations between major depressive disorder, generalized anxiety disorder, fibromyalgia, and myalgic encephalomyelitis/chronic fatigue syndrome
Source: Psychol Med. 2025 Aug 11;55:e232. doi: 10.1017/S0033291725100603 (PMC12370297; doi:10.1017/S0033291725100603)
Supplement: Thomas et al. supplementary material 1 — Thomas et al. supplementary material [file S0033291725100603sup001.docx]

**Supplemental Note 1. Exploratory DWLS Structural Equation Model (SEM) Model Identification and Removal of Covariates**

In order to assess specific unidirectional associations between IDs and FDs that emerged from the analysis of factor scores, we tested three models: (1) a bidirectional relationship between Wave 2/3 MDD and Wave 2/3 FM, (2) a unidirectional model where MDD at Wave 2 predicts FM at Wave 3, and (3) a unidirectional model where the FM at Wave 2 predicts MDD at Wave 3. Common factor means and variances were fixed at zero and one, respectively. Binary variables were modeled by fixing the mean to 0, the variance to 1, and estimating the threshold as a free parameter. Ordinal variables were modeled by fixing the two thresholds to 0 and 1 and estimating the mean and variance as free parameters. Factor loadings were assumed to be invariant over time.

Initially, each common factor was regressed on sex and birth year as covariates. We observed evidence of optimization failure in this series of models. The chi-squared statistic of the FM to MDD model was smaller than the bidirectional model, despite having fewer parameters, see the Table S5.

In the bidirectional model (Figure S9), the standardized coefficients for the autoregressive effect of FM (β= 1.00, 95% CI [0.97, 1.03]) was unity and the effect of MDD2 on FM3 was negative (β= -0.19, 95% CI [-0.23, -0.16]). We suspect this pattern of effects arises out of multicollinearity between FM2 and MDD2 after they are residualized on sex and birth year. A similar pattern of effects was observed when the path from FM2 to MDD3 was omitted from the model. As illustrated in Figure S10, in the FM2 to MDD3 model, the standardized coefficient for the autoregression of FM3 on FM2 was also greater than one (β= 1.04, 95% CI [1.00, 1.08]). In contrast, the FM2 to MDD3 model did not demonstrate this pattern of results, the standardized coefficients were positive and below one; see Figure S11.

Overall, these results are consistent with multicollinearity between the residualized FM2 and residualized MDD2, which prevents stable estimation of the path from MDD2 to FM3, i.e., after adjusting for covariates, MDD2 does not provide additional information about FM3 above the effect of FM2. By contrast, FM2 is a significant predictor of MDD3 in the presence of the autoregression of MDD3 on MDD2. This interpretation is consistent with the results from the model without covariates which indicate that the FM2 to MDD3 model provided the best fit to the data. Thus, covariates were excluded from the analysis that we present in the main text to facilitate model convergence. We performed a sensitivity analysis by re-running the factor score path analysis models excluding covariates to determine if the covariates (sex and birth year) affected the results. Results were nearly indistinguishable when covariates were excluded as illustrated in Figure S12.
